# Diagnostic and therapeutic approach to chronic meningitis in Brazil: a narrative review

**DOI:** 10.1055/s-0042-1758645

**Published:** 2022-12-28

**Authors:** Guilherme Diogo Silva, Bruno Fukelmann Guedes, Ióri Rodrigues Junqueira, Hélio Rodrigues Gomes, José Ernesto Vidal

**Affiliations:** 1 Universidade de São Paulo, Faculdade de Medicina, Hospital das Clínicas, Departamento de Neurologia, São Paulo SP, Brazil.; 2 Universidade de São Paulo, Faculdade de Medicina, Hospital das Clínicas, Departamento de Doenças Infecciosas, São Paulo SP, Brazil.; 3 Instituto de Infectologia Emílio Ribas, Departamento de Neurologia, São Paulo SP, Brazil.

**Keywords:** Meningitis, Diagnosis, Tuberculosis, Meningeal, Meningitis, Cryptococcal, Brazil, Meningite, Diagnóstico, Tuberculose Meníngea, Meningite Criptocócica, Brasil

## Abstract

**Background** Chronic meningitis (CM) is characterized by neurological symptoms associated with the evidence of cerebrospinal fluid pleocytosis lasting > 4 weeks. Studies on the management of CM in Brazil are scarce.

**Objective** To critically review the literature on CM and propose a rational approach in the Brazilian scenario.

**Methods** Narrative literature review discussing the epidemiology, clinical evaluation, basic and advanced diagnostic testing, and empirical and targeted therapy for the most relevant causes of CM. The present review was contextualized with the local experience of the authors. In addition, we propose an algorithm for the management of CM in Brazil.

**Results** In Brazil, tuberculosis and cryptococcosis are endemic and should always be considered in CM patients. In addition to these diseases, neurosyphilis and other endemic conditions should be included in the differential diagnosis, including neurocysticercosis, Baggio-Yoshinari syndrome, and endemic mycosis. After infectious etiologies, meningeal carcinomatosis and autoimmune diseases should be considered. Unbiased and targeted methods should be used based on availability and clinical and epidemiological data.

**Conclusion** We propose a rational approach to CM in Brazil, considering the epidemiological scenario, systematizing the etiological investigation, and evaluating the timely use of empirical therapies.

## Resumo

**Antecedentes** A meningite crônica (MC) é caracterizada por sintomas neurológicos associados à evidência de pleiocitose do líquido cefalorraquidiano por > 4 semanas. Os estudos sobre o manejo da MC no Brasil são escassos.

**Objetivo** Rever criticamente a literatura sobre MC e propor uma abordagem racional no cenário brasileiro.

**Métodos** Revisão da literatura narrativa discutindo a epidemiologia, avaliação clínica, testes diagnósticos básicos e avançados, além da terapia empírica e direcionada para as causas mais relevantes do MC. A presente revisão foi contextualizada com a experiência local dos autores. Além disso, propomos um algoritmo para o manejo da MC no Brasil.

**Resultados** No Brasil, a tuberculose e a criptococose são endêmicas e devem ser sempre consideradas em pacientes com MC. Além destas doenças, a neurossífilis e outras condições endêmicas devem ser incluídas no diagnóstico diferencial, incluindo: neurocisticercose, síndrome de Baggio-Yoshinari e micoses endêmicas. Após etiologias infecciosas, devem ser consideradas a carcinomatose meningeal e doenças autoimunes sistêmicas. Métodos diagnósticos devem ser utilizados com base na disponibilidade, nos dados clínicos e nos dados epidemiológicos.

**Conclusão** Propomos uma abordagem racional para a MC no Brasil, considerando o cenário epidemiológico, sistematizando a investigação etiológica e avaliando o uso oportuno de terapias empíricas.

## INTRODUCTION

Classically, chronic meningitis (CM) is defined as neurological symptoms associated with the evidence of cerebrospinal fluid (CSF) pleocytosis for ≥ 4 weeks. However, in clinical practice, CM can be diagnosed with two separate lumbar punctures or CSF pleocytosis associated with symptoms lasting > 1 month. 1 Chronic meningitis is a relatively uncommon syndrome, corresponding to up to 5 to 10% of all meningitis cases. 2 3

The term CM was defined by Ellner and Bennett in the 1970s 2 . This definition was introduced to separate this syndrome from acute meningitis which presents a duration of symptoms of ≤ 5 days and has pyogenic bacteria and viruses as the main etiologies. On the other hand, subacute meningitis is usually defined when the duration of symptoms is > 5 days and its etiological profile and management is similar to CM. 4 5

## EPIDEMIOLOGY

Globally, but particularly in low- and middle-income countries, CM is usually caused by an infectious agent until proven otherwise. This premise was reported in reviews 2 and documented in case series. 3 6 7

Chronic meningitis has been associated with a myriad of atypical and uncommon etiologies in case reports. Table 1 presents the most important case series with epidemiological information about CM.

**Table 1:** Summary of large case series of chronic meningitis of mixed causes from other countries, divided by country and group (infectious versus noninfectious)

	Country	Infectious causes	Non-infectious causes
Anderson, 1987 ^3^	New Zealand ( *n* = 55)	Tuberculosis 33 Cryptococcal 6 Syphilitic 2 Leptospiral 1 Eosinophilic 4 Herpes 1	Malignant 7 Sarcoid 1
Helbok, 2005 ^6^	Thailand ( *n* = 110)	Cryptococcal 61 Tuberculosis 43 Streptococcus sp 2 Eosinophilic 1	Malignant 3
Erdem, 2017 ^7^	United States ( *n* = 77)	Tuberculosis 37 Syphilis 24 Borrelia 10 Brucella 6	Not included
Total	n = 242	Tuberculosis 113 Cryptococcal 67 Spirochetes 27 Other agents 24 (viruses, parasites, uncommon bacteria)	Malignant 10 Autoimmune 1 (Sarcoid)

The most common infectious disease across studies is tuberculous meningitis. Brazil is a highly endemic country for tuberculosis, 8 therefore, this disease must always be considered in patients with CM. Cryptococcosis comes second, being particularly frequent in people living with HIV (PLWH). Although Sub-Saharan African and Asia concentrate most of the cases of cryptococcal meningitis, in Latin America, we expect ∼ 5,300 cases of cryptococcal meningitis in PLWH each year. 9 In our experience, tuberculosis and cryptococcosis are the most common causes of CM in daily clinical practice in Brazil.

Uncommon bacterial agents are mainly spirochetes ( *Treponema pallidum* , *Leptospira* spp, and *Borrelia burgdorferi* ) *.* Lyme disease is the most common vector-borne disease in the United States and Europe. This disease is mainly caused by the *B. burgdorferi* and is transmitted to humans by certain species of ticks of the *Ixodes ricinus* group. Lyme disease has not been described in Brazil but similar systemic and neurological clinical manifestations were described 10 and named Baggio-Yoshinari Syndrome. Patients with this syndrome showed nonmotile structures similar to those reported as spirochetes in cystic forms identified by electron microscopy analysis in blood samples. The vector of Lyme disease does not exist in Brazil, but the potential transmission of this syndrome occurs via the bites of *Amblyomma* and *Rhipicephalus* genera ticks. *Borrelia burgdorferi* has not been isolated or cultured in our country but authors claim the identification of *B. burgdorferi* *sensu stricto* spirochetes with immunohistochemical methodology using focusfloating microscopy 11 and molecular methods 12 13 Diagnostic criteria for Baggio-Yoshinari Syndrome have been postulated. 14 However, the existence of Baggio-Yoshinari Syndrome is still controversial. Despite this and considering the presence of some cases in clinical practice, if a patient with CM without definite etiology reports consistent epidemiology (i.e. a tick bite episode and/or contact with wild or domestic animals infested with ticks) and shows reactive serologies (ELISA and Western-blotting) for *B. burgdorferi* , the diagnosis of Baggio-Yoshinari Syndrome should be considered, and treatment should be started without delay. Serology results should be evaluated with caution, as cross-reactions have been described within the detection of antibodies to *B. burgdorferi* and *Treponema pallidum* , *Mycobacterium tuberculosis* , *Herpes simplex virus* , HIV-1, HTLV-1, and even some autoantibodies. 15

Non- *Cryptococcus* spp. fungi and parasites are uncommon etiologies of CM. *Candida* spp. and *Aspergillus* spp were described as causes of chronic meningitis worldwide. 5 In Brazil, *Histoplasma capsulatum* , 16 *Sporotrix spp* 17 and *Paracoccidiodes spp* 18 are endemic mycosis that can cause CM.

*Angiostrongylus cantonensis, Gnathostoma spinigerum* and *Baylisascaris procyonis* are the most common causes of infectious eosinophilic meningitis worldwide, especially in Southeast Asia. 19 In Brazil, the most common parasite associated with eosinophilic meningitis is *Taenia solium* , causing neurocysticercosis, particularly in the racemose form. 20 Other parasites that may be observed in cases of CM in our country include *Toxocara* spp 21 and *A. cantonensis* . 22

The most common non-infectious cause of CM is malignant carcinomatosis. In Brazil, delayed diagnosis of cancer is not uncommon, mainly due to limited access to specialized public services. 23 Diagnostic delay may lead to a significant number of patients diagnosed with malignant carcinomatosis without the previous diagnosis of cancer in our country.

Autoimmune diseases are the second most noninfectious cause of CM, particularly neurosarcoidosis. IgG4-related disease is increasingly being recognized as an important cause of chronic meningitis, a knowledge that did not exist at the time of most case series. 24 Data regarding the epidemiology of neurosarcoidosis and pachymeningitis associated with IgG4-related disease in Brazil is scarce. For a simplified list of the major diagnostic groups, see Table 2 .

**Table 2:** Common and uncommon causes of chronic meningitis

Common		Uncommon
**Infectious**	Tuberculosis	Spirochetes(Syphilis, Borrelia, Leptospirosis)
	Cryptococcosis	Intracellular bacterias (Brucella, Bartonella, Nocardia) Uncommon gram positive bacteria (Streptococcus sp) Non cryptococcus fungi (Histoplasma, Candida, Aspergillus) Parasites(Cysticercosis, Toxocara, Angiostrongylus) Viruses(Herpes family, HIV and recently described virus associated with chronic meningitis – e.g., astrovirus)
**Noninfectious**	Carcinomatosis	Sarcoidosis, IgG4-related diseases, Colagenosis (i.e., rheumatoid arthritis)

## CLINICAL EVALUATION

A detailed anamnesis and physical exam should be performed in patients with CM. The most common presenting symptom is headache, occurring in > 80% of patients. Cognitive decline and cranial nerve dysfunction, such as visual loss or ophthalmoplegia, are also important presentations of CM. Absence of fever should not exclude this diagnosis since it is not present in more than half of the cases in some series. 25 Table 3 presents a summary of relevant clinical, epidemiological, cerebrospinal fluid, and neuroimaging information, along with potential corresponding diagnoses. Systemic symptoms may be important to diagnose disseminated disease. Almost half of the patients with neurotuberculosis present lung disease and, hence, the presence of cough and hemoptysis may be a hint. 26 Disseminated fungal disease (e.g., paracoccidioidomycosis 27 or sporotrichoses 28 ) may present mucocutaneous lesions or lymphadenopathy.

**Table 3:** Diagnostic approach to chronic meningitis – initial clinical, cerebrospinal fluid and imaging evaluation

	Investigation	Finding	Potential diagnoses
**Clinical History**	Geographic region of residence/recent travel	Northeastern MG, southern BA	Neuroschistossomosis
		Southest region (SP, PR, RS)	Paracoccidioidmycosis
		Rural areas	Cysticercosis, hystoplasmosis, paracoccidioidomicosis
		Caving	Hystoplasmosis
	Immune status	HIV	Cryptococcosis, tuberculosis, syphilis
		Immunossupressant use	Cryptococcosis, listeriosis
	Socieconomic	Incarcerated	Tuberculosis
		Drinking well water	Cisticercosis
		MSM, sex workers	Syphilis, HTLV, HIV
		Flood and sewer exposure	Leptospirosis
**Investigation outside the CNS**	Skin lesions	Ulcerated nodules	Sporotrichosis, cryptococcosis
		*Eritema nodosum*	Sarcoidosis, tuberculosis, systemic lupus
		*Eritema migrans*	borreliosis
	Ear nose and throat	Chronic sinusitis	Saprophitic fungi (Rhizopus, Rhizomucor and others), granulomatosis with polyangiitis
	Arthritis	Present	Rheumatoid arthritis, borreliosis, sarcoidosis
	Lymphadenopathy	Single, nonsupurative	Lymphoma
		Single, supurative	Tuberculosis, cryptococcosis
		Polyadenopathy	autoimmune disease (lupus, sarcoidosis), leukemia
	Hepatosplenomegaly	Present	Schistossomiasis, lymphoma, leukemia
	Lungs	Migrating nodules (Löefler syndrome)	Schistossomiasis, strongiloidiasis
		Fixed nodules	Sarcoidosis
		Ground-glass opacities	Autoimmune disease
		Miliary nodules	Tuberculosis
	Eyes	Retinal cisticercci	Cisticercosis
		Uveitis	Autoimmune disease, tuberculosis
		Chorioretinitis	Syphilis, cat-scratch disease
**Neuroimaging**	Magnetic resonance or computed tomography of the brain	Basilar enhancement	Tuberculosis
		Mild leptomeningeal enhancement	Autoimmune disease, syphilis, cryptococcosis, tuberculosis
		Pachymeningeal enhancement and thickening	IgG-4-related-disease, sarcoidosis, granulomatosis with polyangiitis
		Meningeal nodules	Sarcoidosis
		Cerebral calcifications, cystic lesions	Cysticercosis
**CSF analysis**	CSF cell count and differentials, protein, and glucose levels	Eosinophilic leukocytosis	Histoplasmosis, strongiloidiasis, schistossomiasis
		Neutrophilic leukocytosis	Early tuberculosis, bacteria
		Very high protein	Tuberculosis
		Mild changes	Autoimmune disease
		Low glucose	Carcinomatosis, tuberculosis, bacteria

Abbreviations: BA, Bahia; CNS, central nervous system; CSF, cerebrospinal fluid; MG, Minas Gerais; MSM, men who have sex with men; PR, Paraná; RS, Rio Grande do Sul; SP, São Paulo.

The epidemiological data should include the geographic region of residence, the presence of a recent history of travel, and the immune status. In Brazil, endemic diseases (i.e., tuberculosis and mycoses) should be considered in the differential diagnosis of CM.

The traveler patient may present several infectious causes. For instance, an individual who traveled to visit caves in Minas Gerais State, Brazil, can be infected by *Histoplasma* spp, whereas an individual who went swimming in a lagoon in the state of Sergipe, Brazil, can be infected by *Schistosoma mansoni* . Besides traveling, other significant exposures include unprotected sexual contact for syphilis or HIV, contact with animals such as rats for leptospirosis, or eating high-risk food such as unpasteurized milk for brucellosis or consumption of snails or raw fishes for angiostrongyliasis. 29 Brucellosis is sporadically described in Brazil, particularly in the Southern Region. 30 Different immunosuppression patterns may predispose to different causes of chronic meningitis 31 : cryptococcal meningitis is the most common cause of chronic meningitis in PLWH and may occur in solid-organ transplant recipients; patients with agammaglobulinemia and those receiving B-cell depleting immunotherapy have a risk of chronic enteroviral meningitis.

## DIAGNOSTIC APPROACH

### Brain neuroimaging

Neuroimaging can be normal or present contrast enhancement in the meninges. When the enhancement occurs in the dura mater, we classify it as pachymeningitis; whereas when the enhancement occurs in the pia or arachnoid, we call it leptomeningitis. 1 The distribution of the meningeal enhancement may help with the differential diagnosis. For instance, for IgG4-related disease, we usually identify the pattern of pachymeningitis and not leptomeningitis.

Classical findings of tuberculous meningitis include prominent leptomeningeal enhancement of the basal cisterns, ventricular dilatation, and vasculitis. 5 32 On the other hand, cryptococcal meningitis includes hydrocephalus and cryptococcomas. The presence of dilatation of Virchow-Robin spaces and mucinous pseudocyst are highly suggestive of cryptococcal meningitis.

However, either unspecific or mild abnormalities are common and immunocompromised hosts usually present with atypical radiological findings. 1 Magnetic resonance imaging (MRI) is preferred over computed tomography (CT) and has the added benefit of increasing the yield of biopsies. 33

### Cerebrospinal fluid: general analysis

Most CM have a CSF with lymphocytic predominance, but other patterns may occur. A study compared CSF characteristics of PLWH with the two most common causes of CM– tuberculous meningitis and cryptococcal meningitis – and neutrophil predominance was significantly more common in patients with tuberculosis (43.8% x 1.8%). 34

Neutrophil predominance may occur in other rare agents such as non-cryptococcal fungal meningitis (e.g., aspergillosis, candidiasis, or endemic mycosis). The presence of more than 10% of eosinophils or 10 eosinophils in the cell count defines eosinophilic meningitis, a context suggestive of parasitic infection. Cerebrospinal fluid protein levels are usually higher in patients with tuberculous meningitis when compared with patients with cryptococcal meningitis in PLWH. Cerebrospinal fluid glucose levels are similar in patients with tuberculous and cryptococcal meningitis in PLWH, 35 whereas CSF glucose levels are lower in patients with tuberculous meningitis when compared with cryptococcal meningitis in individuals with HIV infection. 36 Despite some differences of basic CSF characteristics that appear useful in the differential diagnosis of tuberculous meningitis and cryptococcal meningitis, an accurate algorithm to discriminate these diseases is not possible. This result indicates the need for optimized access to rapid, sensitive, and specific laboratory tests. 34 Abnormalities of basic CSF characteristics cannot differentiate infectious and noninfectious causes of CM such as carcinomatosis, neurosarcoidosis, or rheumatoid arthritis.

### Cerebrospinal fluid: specific analysis

The diagnosis of tuberculous meningitis is difficult. The smear has low sensitivity, and culture can take too long for clinical decision making. Commercial nucleic acid amplification techniques have moderate sensitivity (∼ 60%). 37 Xpert MTB/RIF showed better sensitivity (∼ 80%) for tuberculous meningitis diagnosis, but a negative Xpert MTB/RIF test does not rule out this disease. 38 Higher volume of CSF (> 5 mL) and centrifugation seems to improve the diagnosis performance of Xpert MTB/RIF. 39 Next generation Xpert MTB/RIF Ultra (Xpert Ultra) tests demonstrated greater sensitivity when compared with culture or Xpert MTB/RIF for tuberculous diagnosis. 40 However, its negative predictive value is not sufficiently high to exclude tuberculous meningitis when the result is negative, and as such, it is not a “ruled out” test. 41 The World Health Organization (WHO) recommends the use of Xpert MTB/RIF Ultra as the initial diagnostic test for suspected tuberculous meningitis. 42 43 Furthermore, repeated high volume samples of CSF, preferentially in the first days of hospital admission, are associated with increased diagnostic sensitivity. 44

In contrast to tuberculous meningitis, the diagnosis of cryptococcal meningitis is usually faster, particularly in PLWH, in whom cryptococcosis caused by *Cryptococcus neoformans* complex is frequent. 45 46 Although India Ink stain has low to moderate sensitivity and waiting for culture results can delay treatment, cryptococcal antigen solves this problem as it has high accuracy and fast results. Classically, CSF cryptococcal antigen (CrAg) latex agglutination, a laboratory-dependent test, has been the most used CrAg method. However, CSF lateral flow assay (LFA), a point-of-care test, showed higher accuracy. 47 48 A negative test practically rules out cryptococcal meningitis. In comparison with other antigenic techniques, CrAg LFA requires no pretreatment of sample, presents higher sensitivity for CrAg of all serotypes, is suitable for use in settings with no, minimal, or advanced infrastructure, offers rapid results (∼ 10 minutes), and has a low overall cost. 46 47 48

The diagnosis of cryptococcosis caused by *Cryptococcus gattii* complex is particularly relevant in Brazil, where this fungus is endemic. 49 CrAg LFA uses two monoclonal antibodies impregnated onto an immunochromatographic test strip to detect CrAg for all four *Cryptococcus* serotypes, which is an advantage in comparison with CrAg-latex or enzyme-linked immunoassay. 47 This rationale was confirmed in two studies where the performance of CrAg LFA in HIV-negative patients was high. 48 50 Unpublished result of an ongoing Brazilian study showed a sensitivity of > 90% when CrAg LFA was used in the diagnosis of severe cryptococcosis caused by *C. gattii* in HIV-negative patients (José E. Vidal, personal communication). Currently, the WHO recommends the use of CSF CrAg LFA as the initial diagnostic test for suspected cryptococcal meningitis. 51 Serum and fingerstick CrAg LFA present a high correlation with CSF CrAg LFA and can anticipate the diagnosis of cryptococcal meningitis. 52 Both Xpert MTB/RIF (including Ultra) and CrAg LFA show better performance and were more studied in PLWH and results in other populations should be interpreted with caution. 52 53

For uncommon infectious agents, we have three main strategies: microbiological, immunological, and molecular tests. Repeated CSF cultures for bacteria, mycobacteria, and fungi should be performed in CSF. 5 44 Sampling brain and meningeal tissue with open or stereotactic biopsies may be considered in some cases. 33 When performed, biopsy specimens should always be sent for histological analysis, culture, and, eventually, molecular testing. 33 54

Immunological tests should be performed in blood and CSF, targeting bacteria (i.e., serologies and immunological reactions for syphilis, leptospirosis, BYS, brucellosis, and bartonellosis), virus (HIV-1, enterovirus in B-cell depleted patients), parasites (Western Blot in serum or, less accurate, ELISA in CSF for cysticercosis) and fungi (i.e., immunodiffusion, counterimmunoelectrophoresis, and Western Blot for *Histoplasma* spp, galactomannan for *Aspergillus* spp. and B-D-glucan for *Candida* spp.). 1 6 55

Next-generation sequencing metagenomics is a promising technology to diagnose uncommon agents as it is unbiased and is not directed to a specific pathogen. This technique can be useful in cases of CM without diagnosis and can be performed in CSF and central nervous system samples (brain and/or meninges). Identification of unexpected microorganisms as the cause of CM using metagenomics needs to be ideally confirmed with other techniques. 54

Considering noninfectious causes, we should remember that repeated CSF (up to three high-volume samples) testing improves sensitivity for the diagnosis in meningeal carcinomatosis and MRI of the brain and spinal cord may detect tumoral implants. 56 Considering a metastatic disease, imaging should be directed to the most common sites – breast, lung, and lymphoma in clinical suspicion. 57

For inflammatory causes, we recommend testing for rheumatological panels (i.e., ANA, rheumatoid factor, and ANCA) and searching for typical target organ damage in autoimmune diseases (e.g., malar rash, polyarthritis, and glomerulonephritis). Histological tissue may be necessary to confirm the definitive diagnosis of some causes, as sarcoidosis or IgG4-related disease. 58 59

Clinical (e.g., cough or diarrhea) and laboratorial (i.e., abnormal liver enzymes) systemic evaluation can guide the need of specific studies, as chest or abdomen CT. 1 59 60 However, if the etiology of chronic meningitis cannot be found, systemic images can be performed to detect asymptomatic abnormalities. 5 59

Meningeal and brain biopsy have a diagnostic yield of 30% for chronic meningitis, although it ranges from 5 to 80% according to MRI findings, particularly meningeal enhancement or enhancing lesions; and such gadolinium-positive areas have the highest diagnostic yield in brain/meningeal biopsy. 33

**Figure 1:**
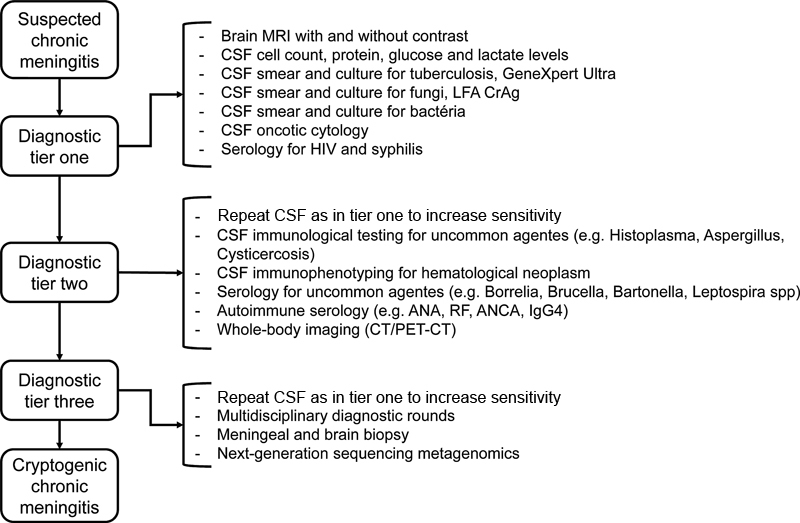
Suggested diagnostic approach for chronic meningitis.

Due to the complexity of some cases of CM, the utilization of a multidisciplinary team of specialists can be necessary, including ophthalmology, infectious disease, rheumatology, radiology, oncology, otorhinolaryngology, and/or neurosurgery. 1 55 Figure 1 represents our suggested diagnostic approach to chronic meningitis. Table 4 lists important diagnostic tests for both targeted and unbiased investigations, ranging from the basic *M. tuberculosis* PCR to metagenomic next-generation sequencing and meningeal biopsy.

**Table 4:** Important diagnostic tests for pathogen identification

**Tests for most common causes of chronic meningitis**
o CSF smear and culture for tuberculosis, *M. tuberculosis* polymerase chain reaction (or GeneXpert Ultra when available) o CSF smear and culture for fungi, LFA CrAg o CSF smear and culture for bacteria o CSF oncotic cytology o Serological testing for HIV and *Treponema pallidum*
**Cerebrospinal fluid analysis and serological testing for uncommon causes**
o Blood and CSF immunological testing for uncommon agents: *Paracoccidoides, Histoplasma, Aspergillus, Cysticercus, Borrelia, Brucella, and Bartonella* – based on epidemiology. o Autoimmune serological testing: ANA, RF, ANCA, IgG4 levels.
**Advanced investigation for undiagnosed cases**
o Multidisciplinary diagnostic rounds o Whole-body imaging (FDG-PET-CT) o Meningeal and brain biopsy o Next-generation sequencing metagenomics

Abbreviations: ANA, anti-nuclear antibodies; ANCA, Antineutrophil Cytoplasmic Antibodies; CSF, cerebrospinal fluid; FDG-PET-CT, fluorodeoxyglucose (FDG)-positron emission tomography *;* LFA CrAg, lateral flow assay for cryptococcal antigen; RF, rheumatoid factor.

## MANAGEMENT

There are two main scenarios for treating CM: targeted therapy for diagnosed etiology or empirical treatment for undiagnosed cases. Even after extensive investigation, approximately a third of CM cases remain unexplained. 55 This investigation is usually extensive and time-consuming, requiring a multidisciplinary approach and noninvasive and invasive diagnostic strategies. Timely diagnosis is challenging, and urgent in a deteriorating neurological patient. For these reasons, empirical therapy is an important and difficult clinical decision.

### Targeted therapy – cryptococcosis and tuberculosis

The two most commonly identified causes of CM, tuberculosis and cryptococcosis, demand prolonged and combined antimicrobial courses associated with adjuvant therapy. The three key principles to the management of chronic tuberculous meningitis are: (1) antituberculous treatment (2 months of rifampicin, isoniazid, pyrazinamide, and ethambutol followed by 10 months of rifampicin and isoniazid) 61 ; (2) evaluation of intracranial pressure and neuroimaging to decide timely neurosurgery, mainly in cases of hydrocephalus; and (3) adjuvant therapy. Steroids have been shown to reduce mortality and should be used in all cases of tuberculous meningitis, independent of the clinical severity. 62

The three key principles to the management of cryptococcal meningitis are: (1) combined antifungal therapy. Induction therapy consists of amphotericin B deoxycholate plus 5-flucytosine for at least 2 weeks followed by a consolidation phase for at least 8 weeks of fluconazole 400–800 mg/day and then a maintenance phase of at least a year of fluconazole 200 mg/day. 45 If available, a lipid formulation of amphotericin should be used due to similar efficacy when compared with amphotericin B deoxycholate but a better safety profile. If 5-flucytosine is not available, high doses of fluconazole (800-1,200 mg/day) can be used. 46 Alternatively, especially when amphotericin is unavailable or intolerable due to toxicity or laboratorial monitoring is limited, 5-flucytosine plus high doses of fluconazole (1,200 mg/day) may be considered 63 (2) aggressive control of increased intracranial pressure. The main initial strategy includes daily lumbar punctures with drainage of 20 to 30 mL, until pressure normalization for > 2 consecutive days. If this intervention is not enough, a ventricular-peritoneal shunt should be obtained in patients with hydrocephalus and lumbar-peritoneal derivation can be placed in patients without hydrocephalus 49 ; (3) evaluation of neuroimaging in order to identify cryptococcomas or parenchymal lesions attributable to cryptococcosis (i.e., mucinous pseudocysts). In these cases, an induction phase of at least 6 weeks is recommended 64 ; 4) supportive care, including administration of intravenous fluids pre- and postinfusion and close electrolyte and kidney function. 46 64 In individuals without HIV or other immunosuppression (e.g., organ transplantation), guidelines recommend at least 4 weeks in the induction phase, mainly because these patients have cryptococcal meningitis caused by *C. gattii* . 65

Adjunctive corticosteroids are not indicated routinely in the treatment of HIV-associated cryptococcal meningitis but can be necessary for the management of paradoxical immune reconstitution inflammatory syndrome. In contrast, corticosteroids can be prescribed in individuals apparently immunocompetent with cryptococcal meningitis due to the presence of important inflammation shown on CSF and neuroimaging. 66

### Empirical treatment in chronic meningitis of unknown etiology

A significant proportion of CM cases remain undiagnosed after extensive investigation, raising doubts about the value of initiating any empirical therapy. Considering that tuberculosis is the most common and clinically relevant cause of CM in some series, repeated CSF, culture, and molecular tests are time-consuming, and negative results do not exclude the diagnosis, empirical treatment for tuberculosis is reasonable in some cases, especially in moderate-to-severe disease. Case series of CM in endemic regions for tuberculosis showed clinical response to empirical treatment in 14 of 28 patients in New Zealand 3 and in 14 of 15 participants in Iran. 67 Another case series from the Mayo Clinic, United States (not endemic for tuberculosis) reported an absence of improvement with this strategy. 25 The different response to tuberculostatics in the United States versus Iran or New Zealand illustrates the importance of considering regional differences and epidemiology in the decision to initiate empirical treatment in patients with CM.

A common doubt in clinical practice is if we should or not add steroids in empirical antituberculosis therapy. On one hand, steroids in tuberculous meningitis are associated with significantly reduced mortality (RR 0.75) 62 ; on the other hand, steroids may be deleterious in other causes, as cryptococcal meningitis in PLWH. 1 In an Iranian case series, undefined CM treated empirically with antituberculosis treatment associated with steroids had a worse prognosis than antituberculosis treatment alone. 67 In Brazil, we consider that after excluding cryptococcal meningitis with a CSF CrAg test, it seems reasonable to initiate antituberculous treatment with steroids, when clinical and paraclinical data support a high pretest probability of tuberculosis (i.e., compatible CSF abnormalities, typical neuroimaging, or compatible chest CT).

As cryptococcus is as important as tuberculosis in some series of CM, 6 empirical treatment with antifungals can be considered in clinical practice. However, unlike tuberculosis, the diagnosis of cryptococcal meningitis is usually straightforward. In addition, amphotericin is associated with significant morbidity. For these reasons, empirical treatment of cryptococcosis is largely unjustified. However, other noncryptococcal, but amphotericin-responsive fungal infections occasionally cause CM (particularly histoplasmosis and, rarely, candida, paracoccidioidomycosis, and sporotrichosis). 68 69 70 Hence, after an empirical nonresponsive use of medications to *M. tuberculosis* and other bacterial infections, a trial with amphotericin could be considered. In patients with CM and severe neurological manifestations (i.e., in intensive care unit), after excluding cryptococcal meningitis, immediate empirical treatment with antibiotics and tuberculostatics should be prescribed. The decision to add antifungal therapy must be decided on an individual basis.

Unexpected agents can be identified. One study reported 21 patients with undiagnosed CM that responded to penicillin. Bacterial infections caused by penicillin-sensitive organisms related to chronic meningitis include syphilis, borreliosis, leptospirosis, brucellosis, nocardiosis, actinomycosis, listeria, and some species of streptococcus. 71 Penicillin is a possible empiric therapy as it covers a broad spectrum of bacteria related to CM and is a well-tolerated drug. Ceftriaxone is an alternative for some of these agents. Unfortunately, increasing resistance to penicillin reduces the efficacy of this approach. Viruses and parasites are rare causes of CM, and this fact justifies not starting an empirical treatment.

**Figure 2:**
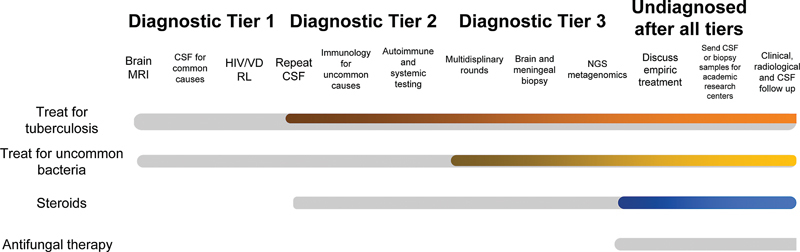
Suggested therapeutic approach for chronic meningitis of unknown cause.

Chronic meningitis can also be noninfectious. After excluding neoplastic meningitis, noninfectious causes are mainly inflammatory – sarcoidosis, IgG4-related disease, or meningitis associated with rheumatological diseases (i.e., systemic lupus erythematosus or rheumatoid arthritis). That is why steroids are also a logical empirical treatment. Both in the United States and New Zealand, 3 25 prednisone resulted in clinical improvement in a significant number of patients with undefined CM. Consequently, after reasonable exclusion of common infectious causes, steroids could be considered. Figure 2 shows our suggested treatment approach to CM.

We emphasize that our recommendations must be weighted with the individual assessment of each case, and medications may be prescribed in a case without definite diagnosis guided by epidemiological, clinical, and laboratory findings or exclusions. For example, even in areas where tuberculosis is common, a trial with steroids alone may be prioritized when the patient with CM is clinically stable and the neuroimaging and systematic findings are suggestive of IgG4-associated pachymeningitis. In addition, in scenarios where tuberculosis is uncommon, treatment with steroids alone, with follow-up clinical and neuroimaging is a reasonable approach to cases of CM for which no diagnosis can be established despite extensive evaluation. 31

In conclusion, tuberculosis and cryptococcosis are endemic in Brazil and should always be considered in patients with CM. However, uncommon causes are frequently treatable. Uncommon causes endemic in Brazil include neurocysticercosis and endemic mycosis. Carcinomatosis is the most common noninfectious cause, but steroid-responsive diseases (i.e., sarcoidosis or IgG4-related disease) should not be overlooked. Strategies to improve management are: (1) choose the best available tests for common causes (i.e., Gene Xpert Ultra for tuberculosis and LFA for cryptococcosis); (2) targeted investigation of specific causes based on clinical and epidemiological clues; (3) consider unbiased strategies for uncommon agents (i.e., next-generation sequencing metagenomics); (4) multidisciplinary case discussion (i.e., neuroradiology, infectious disease, rheumatology, neurology, neurosurgery) to define the best tools, including brain and meningeal biopsy; and (5) a systematic approach to CM of unknown cause.
